# Robotic Surgery: The Future of Gynaecology

**DOI:** 10.7759/cureus.30569

**Published:** 2022-10-21

**Authors:** Isha Chandrakar, Sandhya Pajai, Shikha Toshniwal

**Affiliations:** 1 Department of Obstetrics and Gynaecology, Jawaharlal Nehru Medical College, Datta Meghe Institute of Medical Sciences, Wardha, IND

**Keywords:** cervical cancer, robotic myomectomy, robotic hysterectomy, da vinci xi, zeus

## Abstract

Robot-assisted surgery is the next phase in the process of transhumanism. Presently, robotic surgery is used in various benign and malignant gynaecological procedures. Robot-assisted surgery is significantly superior to open surgeries in post-surgical hospital stays; however, the difference is less significant in the case of laparoscopic surgery. Estimated blood loss in robotic surgery may be less. Regarding postoperative time, the results have been inconsistent due to variations in surgeons' experience. The primary drawbacks of robotic systems are their high installation and maintenance costs and lack of tactile feedback. Though robotic surgery allows easy dissection and fine suturing and has a faster recovery rate, to decide whether it should become the mainstream of gynaecological procedures, more randomized controlled trials are needed.

## Introduction and background

**Figure 1:**
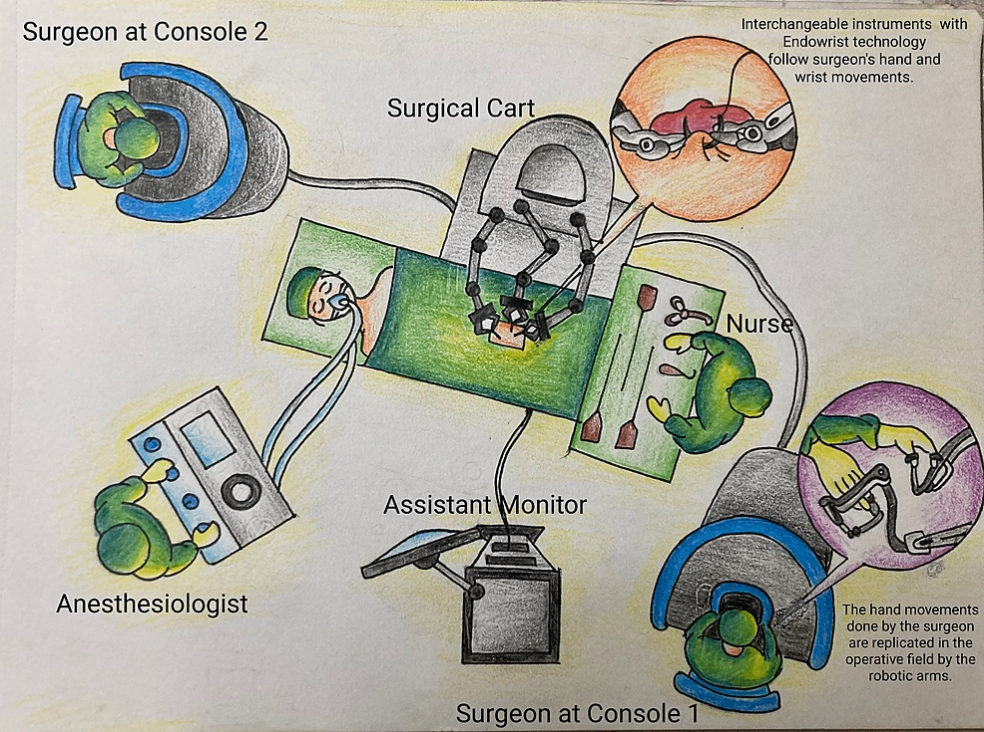
Operation theatre setup for robot-assisted surgery. Illustrated by Isha Chandrakar. Modified with permission from Copyright ©️ 2022, Intuitive Surgical Operations, Inc.

Some of the greatest advances in robotic surgery technology were inspired by the National Aeronautics and Space Administration (NASA) [1]. Robotic application to surgery was started in the 1970s as a military project approved by NASA and funded by Defense Advanced Research Projects Agency (DARPA) as a replacement for the surgeon's physical presence and providing care to astronauts and soldiers on the battlefield [2]. The first robotic surgery was performed in Canada in 1983 by an orthopaedic surgeon and his team, and the robot was named Arthrobot [1]. In 1958, Kwoh and his team performed a brain biopsy under computed tomography (CT) guidance with the assistance of a robotic arm-PUMA560.1 [3]. Robotic surgery evolved through PROBOT (first prostate surgical robot), ROBODOC (first hip replacement surgical robot), ZEUS Robotic Surgical System (ZRSS), and Da Vinci Surgical System [1]. The first-generation robots were used in performing image-guided precision tasks. The second and current generation robots are designed in a master-slave configuration, in which the surgeon (master) controls the robotic arms (slave) with multiple degrees of freedom [4]. The first robotic gynaecological procedure was performed by the ZRSS to reconnect fallopian tubes in Cleveland, Ohio, United States (US). In 2009, the Da Vinci system was launched in Israel. The manufacturing of the ZRSS was discontinued in favour of the Da Vinci system, which is equipped with a compact platform moving on wheels, three to four robotic arms, and an immersive stereoscopic camera giving a 10-fold magnified view [4]. The latest generation of the Da Vinci Xi system has been upgraded with features like near-infrared technology, less bulky, thinner arms, and arranged more ergonomically, enabling multi-quadrant procedures [4]. Figure 1 depicts the operation theatre arrangements for robotic surgery.

The field of gynaecology continuously offers various opportunities for ingenious surgical methods and the development of pre-existing techniques. Since the Da Vinci surgical system got approval from FDA, over 6730 Da Vinci robotic systems have been installed across the world and over 10 million robotic surgeries have been executed worldwide [5]. The Da Vinci surgical system is used to treat benign gynaecological conditions like fibroids, endometriosis, ovarian growths, pelvic prolapse, and abnormal menses, or for malignancies such as endometrial cancer, cervical cancer, or ovarian cancer [1]. In 2011, in the US, around 27% of hysterectomies performed for benign conditions were robot-assisted [6].

In 2006, the All India Institute of Medical Sciences (AIIMS), New Delhi, India, got its first urological robotic installation. As of July 2019, India has 66 centres with 71 robotic installations and over 500 trained robotic surgeons [7]. In these years, over 1400 surgeries have been performed using the robotic system. Dr Sudhir P. Shrivastava developed India's first indigenous and cheapest general robotic surgery system, MANTRA, which has completed its first human clinical trial study with 18 complex abdominopelvic procedures at Rajiv Gandhi Cancer Institute of India and is expected to hit the markets by March 2023 [8,9]. The trend suggests that the rise of robotic surgery in India will be huge.

The aim of this review is to summarize the assets and liabilities of robotic surgery and various studies and literature related to robot-assisted surgery in gynaecology.

## Review

Advantages and disadvantages of the robotic system

The advantages of minimally invasive surgeries like a shorter post-surgical hospital stay, less projected blood loss, and lower patient morbidity compared to open procedures have also been observed here [10]. It provides a three-dimensional (3D) view and thus a better display of the surgical field and augmentation of surgical instruments to seven degrees of freedom. It permits tremor-free handling of the instruments, adding to the surgery's precision and finesse, allowing finer suturing and dissection of poorly accessible tissues and decreasing the surgeon's work fatigue, especially during long and complex procedures. The Da Vinci Xi allows varying use of optics in all the trocars. Robotic surgery has been criticized for using larger trocars compared to laparoscopy; this has now been avoided using smaller trocars, resulting in smaller scars, and is therefore cosmetically better [11]. Another advantage is less postoperative pain and less need for analgesics [12,13].

The drawbacks of the robotic system include the high price of installation and maintenance, extended operating hours, especially in the beginning, and additional time for docking and learning curve. Doctors and nursing staff must be adequately trained to work with robotic systems [14,15]. Another major disadvantage is the absence of tactile feedback. The advantages and limitations of robotic surgery are shown in Table 1.

**Table 1:** Advantages and disadvantages of robotic surgery *Intuitive Surgical, Inc., Sunnyvale, California, United States

Advantages	Disadvantages
Ergonomics	High cost of disposable materials and its maintenance.
Intuitive handling of instruments.	Absence of tactile feedback.
3D optics without additional equipment.	Additional learning curve.
7 degrees of freedom.	Additional time for docking.
Faster learning curve.	
Digital networking.	
Dual console	
Integrated fluorescence visualization (Firefly® System*)	
Less postoperative pain.	

Robotic surgery in benign gynaecological diseases

Robotic surgery can be employed in a majority of gynaecological diseases. However, due to its high cost, it is unlikely to become a routinely used procedure in treating benign gynaecological conditions. Nevertheless, it is necessary to develop various innovative techniques to make robot-assisted surgery feasible in treating benign gynaecological diseases. Various uses of robotic surgery in benign gynaecological conditions are shown in Table 2.

**Table 2:** Uses of robotic surgery in benign gynaecological conditions

Condition	Commonly used robot-assisted surgical procedure
Endometriosis	Total abdominal hysterectomy
Uterine fibroids/leiomyoma	Myomectomy
Pelvic organ prolapse	Sacrocolpopexy
Re-fertilization	Fallopian tube reanastomosis
Abnormal uterine bleeding	Hysterectomy
Pelvic pain	Hysterectomy

Myomectomy 

Myomectomy is the removal of the most common benign growths in the uterus, i.e., leiomyomas/uterine fibroids. The use of the robotic approach has increased in popularity as suture-intensive surgery and robotic arms make suturing simple and easy [4]. Several studies contrasted robot-assisted myomectomy with laparoscopy and open surgeries. In one of the studies, the size of the myoma was the main focus [16], and the other consisted of a more general view of the surgery and its result [17]. Both robotic and laparoscopic myomectomies had the same operative times [18-20]. Open myomectomy consumed less time than robot-assisted myomectomy [18,21]. According to Bident et al., the calculated blood loss in robotic surgery was 100ml, and that with laparoscopic surgery was 250ml [19]. Compared to open myomectomy (437.5 ml), the loss was notably low in robot-assisted surgery. According to Bident et al., the duration of post-surgical hospital stay was the same in both laparoscopic and robotic surgery [19]. When contrasted with the open technique, Ascher-Walsh et al. and Barakat et al. reported that the duration of postoperative hospital stay was significantly shorter in the case of robotic myomectomy (0.5 day vs 3.28 days and one day vs three days, respectively) [18,21].

A study conducted by Gunnala et al. concluded that myoma size should not be a factor taken into consideration while performing a robotic myomectomy and, also, the patient may be discharged on the same day of the surgery [16]. A seven-year retrospective study was conducted by Sangha et al., which included 310 women who underwent myomectomy; Of these, 40% wanted pregnancy [22]. Of the women who wanted pregnancy, 40% conceived, out of which 61% delivered a viable child in their first pregnancy, 6% in their second pregnancy, and 10% women delivered a second viable child. The study concluded that there was no effect of surgical technique, the number of uterine incisions, age, or race of the patient on the incident or outcome of pregnancy. Thus, myomectomy done to preserve fertility resulted in nearly 25% live births, irrespective of the surgical technique used [22]. 

In conclusion, robot-assisted myomectomy is superior to laparoscopic and open myomectomies regarding recovery rate, suturing skills, and rate of morbidity [17]. The effect of robotic surgery on fertility is similar when contrasted to both laparoscopic and open myomectomies [22]. 

Hysterectomy 

The surgical removal of the uterus is referred to as hysterectomy. Along with the uterus, the cervix, ovaries, fallopian tubes, and other surrounding structures are also removed if indicated. It is a commonly performed surgery across the world. Hysterectomy's indications are often benign, such as uterocervical or vaginal prolapse, chronic pelvic pain, abnormal uterine bleeding, and uterine fibroids. In many developed countries, robotic hysterectomy is the alternative to conventional laparoscopic surgeries. In the US, the repeated employment of robotic hysterectomies has decreased the incidence of abdominal (open) hysterectomies. Robotic surgeries are well suited for severe obesity, large uteri and adhesions [4]. 

According to Lim et al.'s study, robotic hysterectomies were associated with lesser intra- and postoperative complications than vaginal and abdominal (open) hysterectomies [23]. Swenson et al. found that postoperative complications, surgical site infections, and the need for blood transfusions were lower with robotic hysterectomies [24]. As per Landeen et al., the operating times in the robotic and laparoscopic hysterectomies were similar (117.2 mins vs 118.3 mins) [25]. According to Swenson et al. and Sarlos et al., robot-assisted surgery was more time-consuming than laparoscopic hysterectomies (2.3 hours vs 2.0 hours according to Swenson et al., and 108.9 mins vs 82.9 mins according to Sarlos et al.) [24,26]. Swenson et al., Landeen et al., and Sarlos et al. also reported that the calculated blood loss and duration of post-surgical hospitalization were less with robotic hysterectomy [24-26].

In conclusion, robotic hysterectomy is superior to laparoscopic hysterectomy in terms of low intra- and postoperative complications, less blood loss, and short duration of post-surgical hospital stays [23-26]. Many gynaecologists with less experience in conventional laparoscopy are eager to embrace robotic surgery [4].

Sacrocolpopexy

Urogynaecological procedures can be performed safely, rapidly and precisely with robotic arms. Sacrocolpopexy (SCX) is a surgical method for treating pelvic organ prolapse at a young age, which occurs due to the weakening of the normal support of the pelvic floor. It is a commonly performed surgery for the suspension of vaginal prolapse. Various studies were conducted regarding the use of robotic SCX for managing vaginal vault prolapse, the effect of obesity on robotic SCX, and the single port technique [1].

According to Paraiso et al.'s randomized control trial (RCT), total surgical time and suturing time were shorter with robotic SCX as compared to laparoscopic SCX (119 min vs 265 min and 63 min vs 98 min, respectively), whereas post-surgical hospital stay was almost the same in both the procedures (1.79 days vs 1.41 days) [27]. Awad et al. and Geller et al. conducted studies to compare laparoscopic and robotic SCX and found that estimated blood loss was lesser with robotic SCX [28,29]. Elliot et al. conducted a cost-effectiveness study and contrasted robotic, laparoscopic, and open SCX surgeries and reported that robot-assisted SCX was the most expensive [30].

Robotic Single-Port SCX: Matanes et al. assessed the single port SCX, in which the surgeon gave a single incision resulting in a small scar and concluded that it is an achievable technique with minimum loss of blood, fewer complication rates, quicker recovery rate, lesser postoperative hospital stays, lesser postoperative pain, and virtually scar-free results [31]. Though robotic SCX has many benefits over conventional laparoscopy, its high cost cannot be overlooked.

Endometriosis

In endometriosis, the tissue lining the uterus, i.e., the endometrium, grows outside the uterus. It is a common gynaecological condition affecting 2-10% of women of childbearing age. Laparoscopic surgery for endometriosis is challenging for gynaecologists due to dense adhesions, loss of adnexal function, and poor reproductive outcomes. Robotic surgery can make this task easier by providing a magnified 3D surgical field and easy removal of the adhesions and fine suturing. However, the assets of robot-assisted surgery over laparoscopy in treating advanced endometriosis remain uncertain [1].

A meta-analysis was conducted by Chen et al. to assess the safety and effectiveness of robotic surgery compared to laparoscopy for managing advanced-stage endometriosis [32]. They observed no distinctness in calculated blood loss, intra- and postoperative complication rates, and postoperative hospitalization.

Reanastomosis of the Fallopian Tube

Reanastomosis of fallopian tubes for the purpose of re-fertilization has a success rate of 67.6% with the open approach and a 5.6% chance of ectopic pregnancies [33]. In contrast to conventional laparoscopy, the robotic approach provides more delicate handling of structures and precise suturing. The disadvantage is a longer operating time. The advantages include shorter post-surgical hospitalization [34].

Robotic surgery in malignant gynaecological diseases

The application of robotic surgery for malignant gynaecological indications shows a rising trend. Table 3 shows the potential uses of robotic surgery in malignant gynaecological conditions.

**Table 3:** Uses of robotic surgery in malignant gynaecological conditions

Malignant condition	Commonly used robot-assisted surgical procedure
Cervical cancer	Radical hysterectomy, total mesometrial resection (TMMR).
Endometrial cancer	Hysterectomy, peritoneal mesometrial resection (PMMR).
Lymphadenectomy and sentinel biopsy (dye and fluorescence)	Pelvic lymphadenectomy, paraaortic lymphadenectomy.
Ovarian cancer	Staging of early ovarian carcinoma, de-bulking advanced ovarian carcinoma, omentectomy

Endometrial Cancer

Endometrial cancer begins in the cell layer forming the lining of the uterus (endometrium). It is a hyperestrogenic condition. A typical indication of robotic surgery in gynaecology oncology is endometrial cancer. Minimally invasive surgery is the accepted standard of care for endometrial cancer surgical staging [2].

Robotic Surgery Vs Laparoscopic Surgery: Barrie et al. conducted an observational study and reported that robotic hysterectomy with or without pelvic or para-aortic lymph node clearance had a shorter surgical time [35]. It was observed that hysterectomy without lymph node dissection took 125 min vs 136 min, and hysterectomy with pelvic or para-aortic lymph node dissection took 186 min vs 244 min. A study conducted by Mäenpää et al. observed a shorter operative time in robotic surgery as compared to the laparoscopic approach and concluded that robotic surgery is a safe and effective alternative in managing endometrial cancer surgically [36]. Many studies reported that the calculated blood loss with robotic surgery was notably lesser. When contrasted with the number of lymph nodes dissected, some studies showed robotic surgery superior, some reported inferior, while some found no significant difference [1].

Robotic Surgery Vs Open Surgery: Many studies showed robotic surgery superior in calculated blood loss and postoperative hospitalization. In their study, El Sahwi et al. found a shorter operative time for robot-assisted surgery and Hindshaw et al. showed a similar operative time [37,38]. Some studies found that robotic surgery is superior in the clearance of lymph nodes, a few noted robotic inferiority, whereas some showed no difference. Though robotic surgery is expensive as compared to open surgeries, after stratification and a thorough review of the postoperative hospital costs in open surgery when compared to robotic surgery, it is concluded that robot-assisted surgery is less expensive than open surgery [39,40].

Robotic Single-Port Surgery: A study was conducted by Corrado et al. to evaluate the practicality and safety of robot-assisted single-port hysterectomy with or without pelvic lymph node clearance, which included 125 patients [41]. Total operative times reported were 122 min. The calculated blood loss was 50 ml. Of the total patients, 16.8% underwent pelvic lymph node removal and 13 lymph nodes were cleared. Post-surgical hospital stay ranged from one to three days, and no intraoperative complication was reported. One of the patients was transformed into vaginal surgery due to pulmonary morbidity. The study concluded that the robotic approach is safe and feasible for grading disease and can become the management of choice in patients having International Federation of Gynecologists & Obstetricians (FIGO) stage 1-2 endometrial cancer [41]. However, more RCTs are needed to support these results.

Cervical Cancer

Cervical cancer is a global problem. The most common is squamous cell carcinoma, followed by adenocarcinoma. For patients having FIGO stage 1A2-2A cervical cancer, radical abdominal hysterectomy has been the treatment of choice. The laparoscopic approach has been performed in small numbers due to its complexity. Robotic surgery offers fewer challenges and can become an ideal surgery. Compared to abdominal and conventional laparoscopic radical hysterectomies, robotic surgery ensures lesser blood loss, shorter surgical times, shorter postoperative hospitalization, and lower complication rates [10]. 

Soliman et al. conducted a comparative study and concluded that calculated blood loss was significantly low with robot-assisted surgery (115.5 ml) as compared to radical laparoscopic hysterectomy (171 ml) [42]. Seven studies showed that radical robotic hysterectomy ensured shorter post-surgical hospital stay ranging from 1-3.7 days and 2.8-5 days for open surgery. All seven studies also reported lower calculated blood losses [42-48]. Cantrell et al. showed no marked distinctness in the overall survival rate following radical hysterectomy, whether done via robotic, laparoscopic, or open approach [49]. No difference in postoperative chemotherapy or radiotherapy was found [49].

A non-RCT was performed by Gallota et al. in stage 1B2-3 cervical cancer patients who had a robot-assisted radical hysterectomy with pelvic or para-aortic lymph node dissection within six weeks of chemotherapy or radiotherapy to evaluate the feasibility and complication of the procedure [50]. The study concluded that the procedure was a success in 97.5% of patients. The median surgical time reported was 185 mins. Calculated blood loss was 50-300 ml, and the duration of hospital stays post-surgery ranged from one to four days. There were no intraoperative complications. In about 30% of patients, complications were seen, and recurrence was observed in 12.5% [50].

Ovarian Cancer

Ovarian cancer is the growth of cells that form in the ovaries. Ovarian cancers are not very common. The robotic approach to ovarian cancer is still debated, as published data are scarce. A study by Feuer et al. to assess the surgical outcome in 63 patients having ovarian cancer who underwent robot-assisted surgery reported longer surgical time, less calculated blood loss, and shorter postoperative hospitalization [51]. No difference was reported in the complication rates, lymph node retrieval, and recurrence rate between the robotic and laparoscopic surgery.

To date, robotic surgery for ovarian cancers is performed for diagnostic purposes. A significant advantage of the Da Vinci Xi system is the ability to perform multi-quadrant surgery, which can enhance robotic surgery in patients with ovarian cancer in the next few years.

## Conclusions

The 3D magnified surgical field, better ergonomics, tremor-free handling, seven degrees of freedom, and the fine dissection and suturing offered by robotic surgery may help switch from open surgery to minimally invasive surgery. In various aspects like lesser estimated blood loss, shorter post-surgical hospital stays, and lower intraoperative and postoperative complications, robotic surgery is superior to open and laparoscopic surgeries in treating both benign and malignant gynaecological conditions. On the other hand, in terms of surgical times, the results are inconsistent and this may be due to variations in surgeon experience. Though robotic surgeries are expensive as of now, the introduction of alternative manufacturers will drastically transform the cost situation. The fact that the robotic approach complements but does not replace conventional laparoscopic surgery is undisputed.
